# Supplementary material for: The mouth of America: the oral microbiome profile of the US population
Source: medRxiv. 2024 Dec 7:2024.12.03.24318415. Preprint. [Version 2] doi: 10.1101/2024.12.03.24318415 (PMC11643230; doi:10.1101/2024.12.03.24318415)
Supplement: Supplement 1 [file media-1.docx]

**Data supplement index for: “The mouth of America: the oral microbiome profile**

**of the US population.”**

| **Item** |
| --- |
| 1. Supplemental methods (DNA extraction, sequencing, bioinformatics, and quality control) |
| 1. Figures S1 and S2: relative abundance of major phyla and genera in quality control samples across sequencing plates/batches |
| 1. Figure S3: Scree plot of principal coordinate analyses (PCoAs) for beta diversity matrices (Bray-Curtis, unweighted UniFrac, and weighted UniFrac) |
| 1. Figures S4-S6: Results of associations of covariates with alpha diversity metrics (Faith’s PD, Shannon-Wiener Index, and Simpson Index) |
| 1. Figure S7: Heatmap of associations of covariates with prevalence and relative abundance of genera |
| 1. Table S1: Taxonomy key |
| 1. Tables S2-S6: Prevalence at phylum (L2), class (L3), order (L4), family (L5), and genus (L6) levels |
| 1. Table S7: Characteristics of NHANES 2009-2012 participants aged 18-69 years with or without oral microbiome data |
| 1. Tables S8-S11Taxa x taxa correlations of relative abundance at phylum (L2), class (L3), order (L4), and family (L5) |
| 1. Table S12: S11Taxa x taxa correlations of relative abundance at gen(L6) level |
| 1. Table S13: Code and variable definitions used in the analyses |
| 1. Tables S14: Associations of covariates with first 100 principal coordinates for beta diversity matrices (Bray-Curtis) , unweighted UniFrac, and weighted UniFrac) |
| 1. Tables S15: Associations of covariates with first 100 principal coordinates for beta diversity matrices (unweighted UniFrac) |
| 1. Tables S16: Associations of covariates with first 100 principal coordinates for beta diversity matrices (weighted UniFrac) |
| 1. Table S17: Associations of covariates with prevalence of genera, logistic regression |
| 1. Table S18: Associations of covariates with relative abundance of genera, Poisson regression |
