# Supplementary figures and images for: The mouth of America: the oral microbiome profile of the US population
Source: medRxiv. 2024 Dec 7:2024.12.03.24318415. Preprint. [Version 2] doi: 10.1101/2024.12.03.24318415 (PMC11643230; doi:10.1101/2024.12.03.24318415)

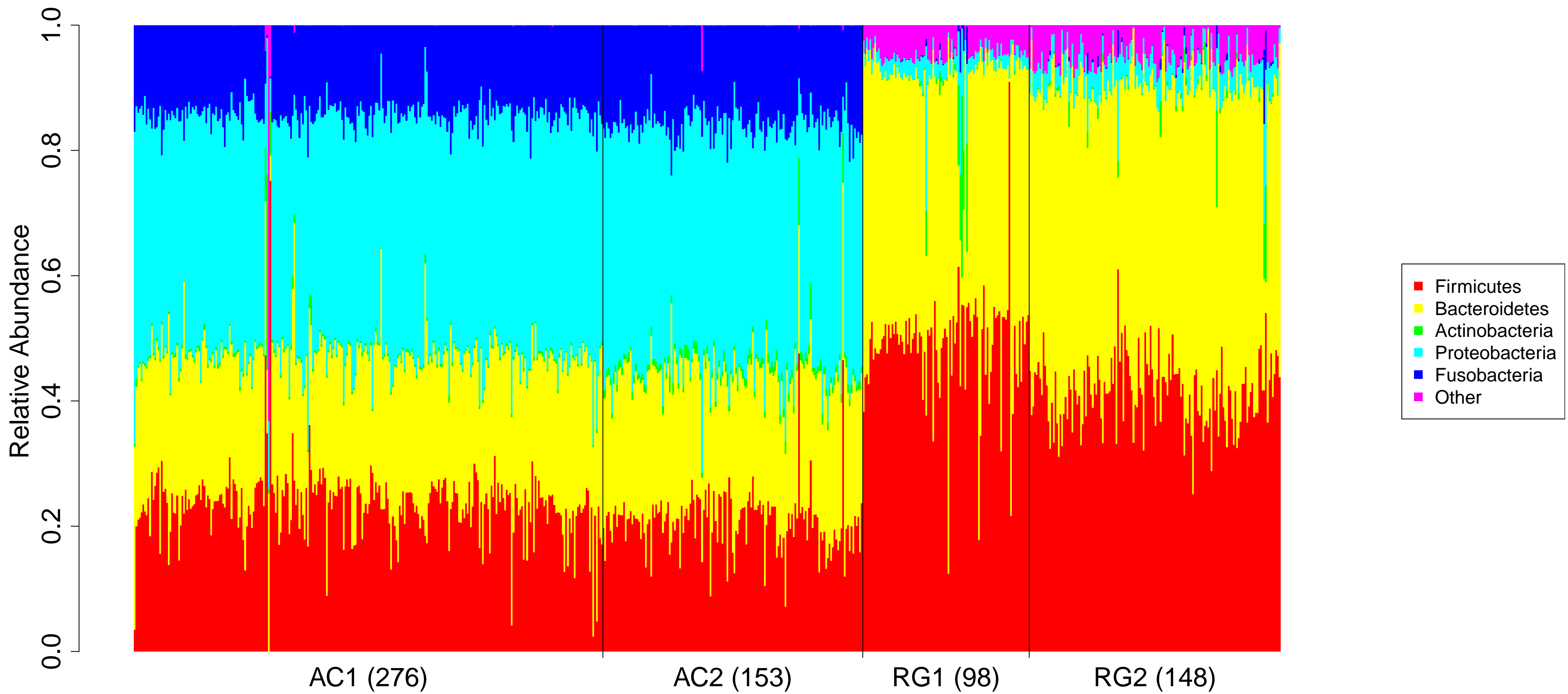

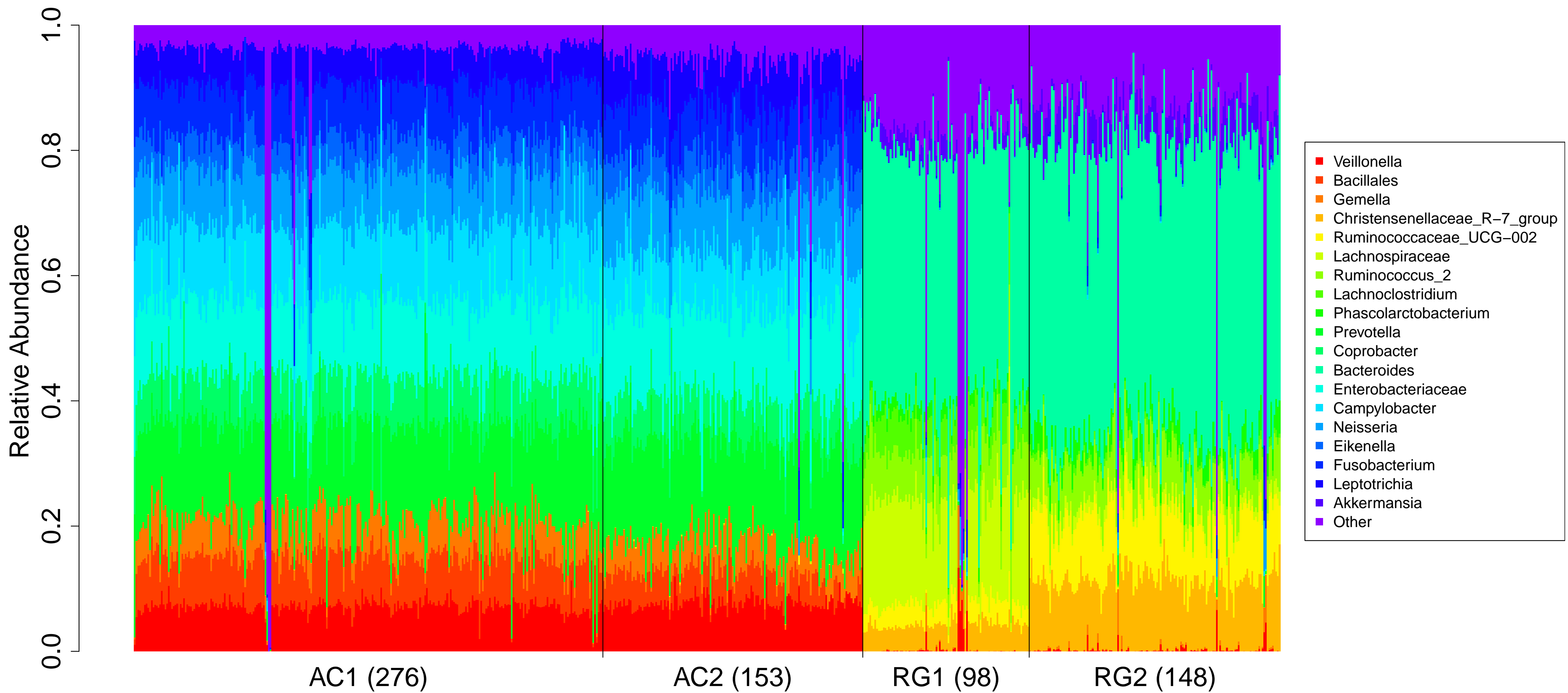

Supplement: Supplement 14 [file media-14.pdf]

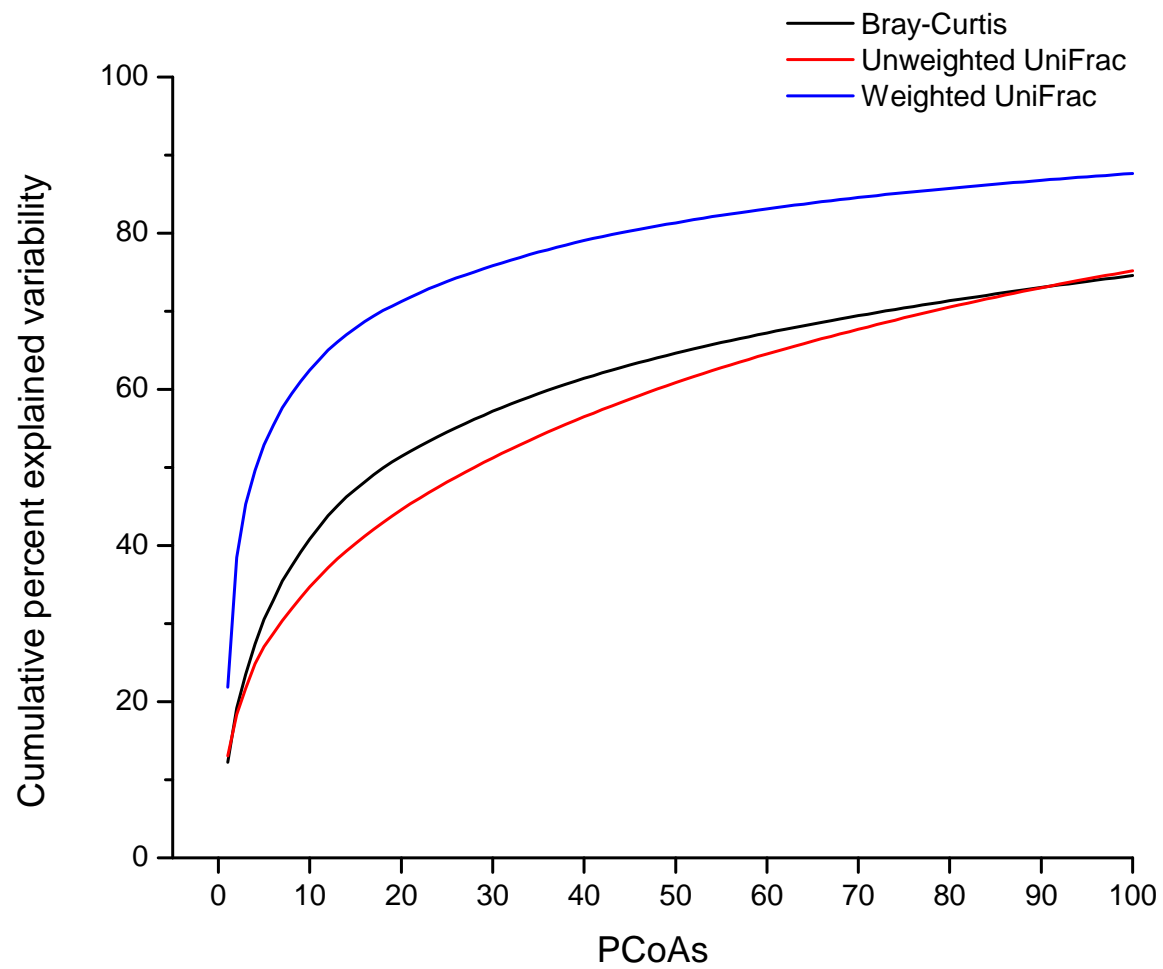

Supplement: Supplement 15 [file media-15.pdf]

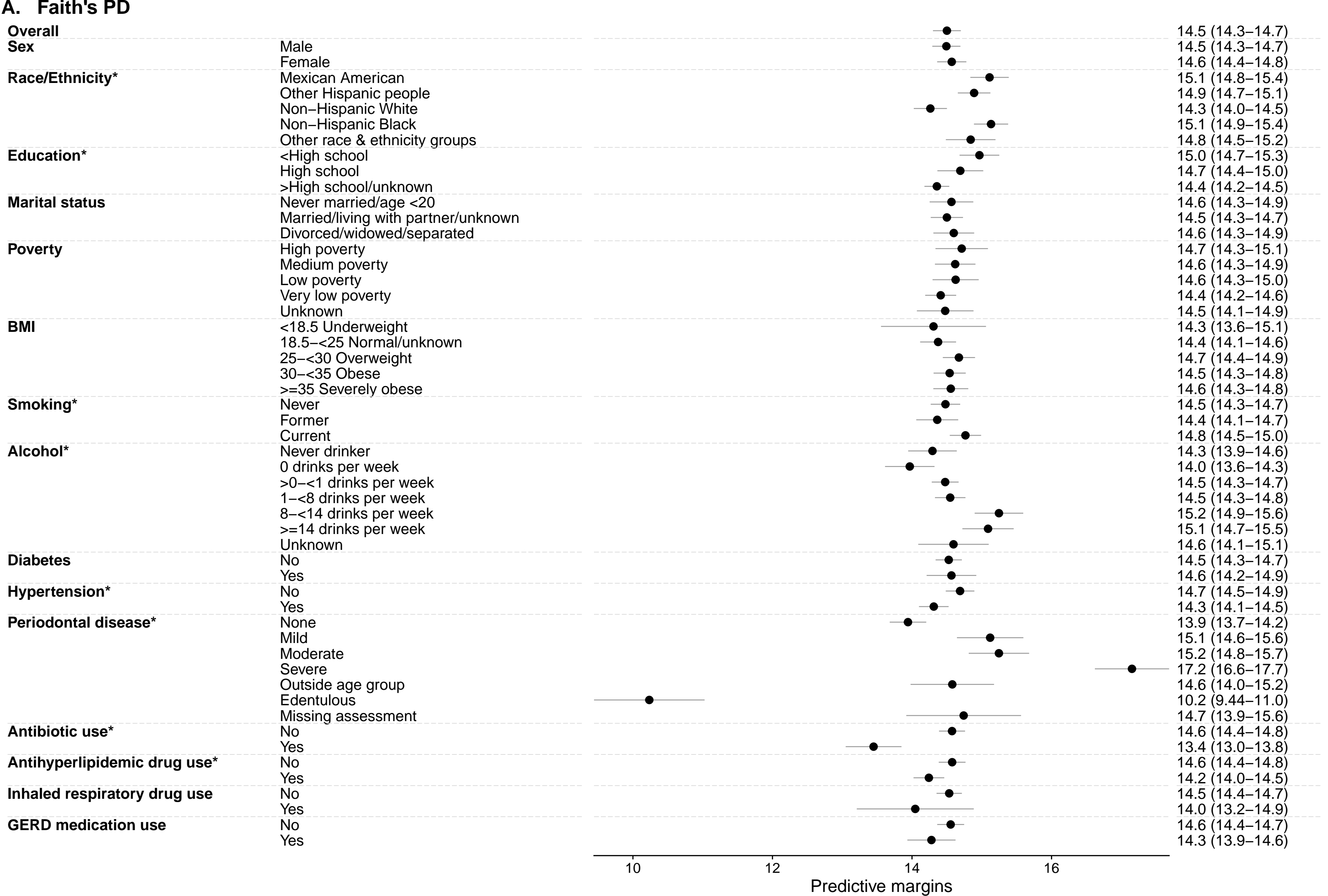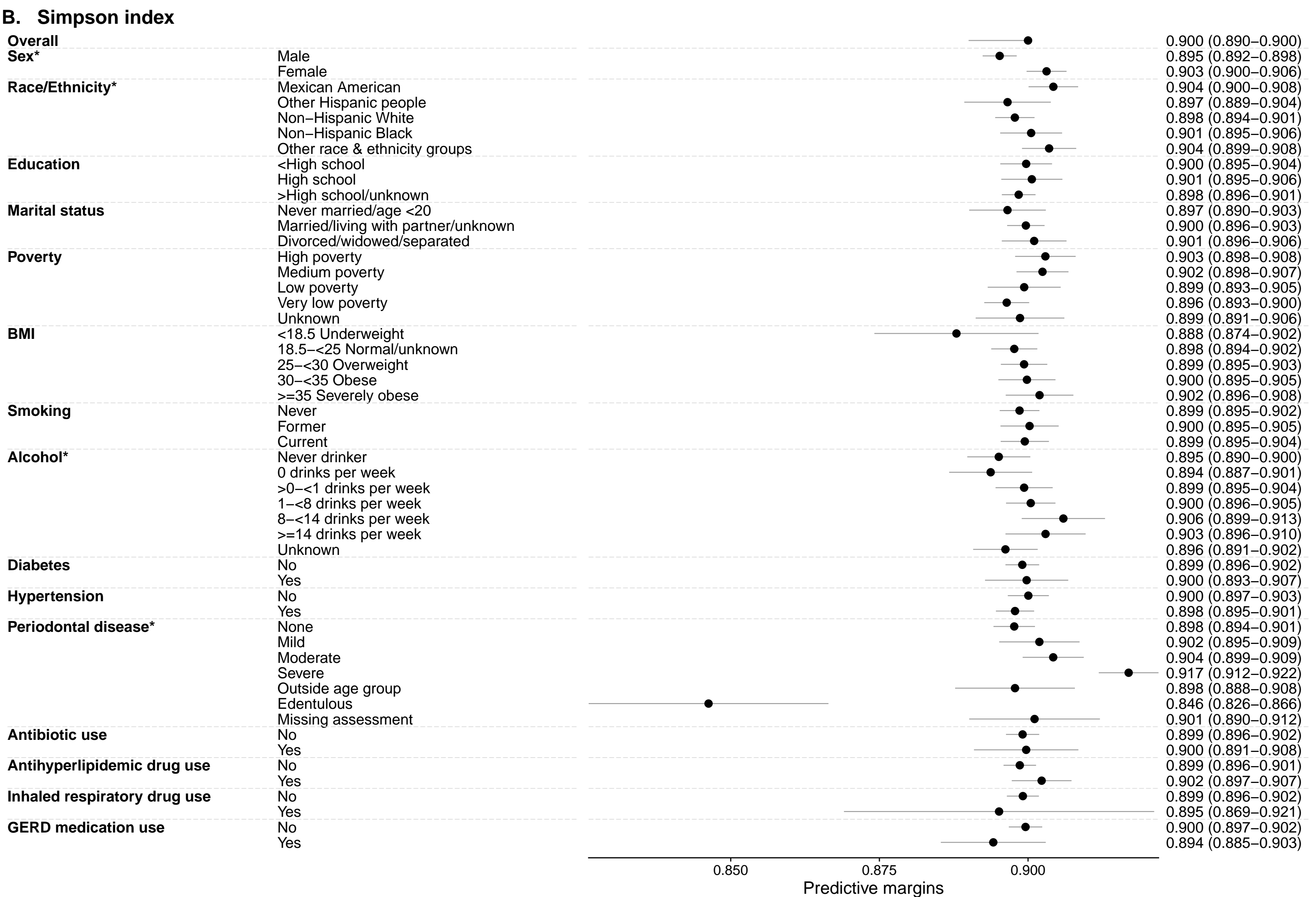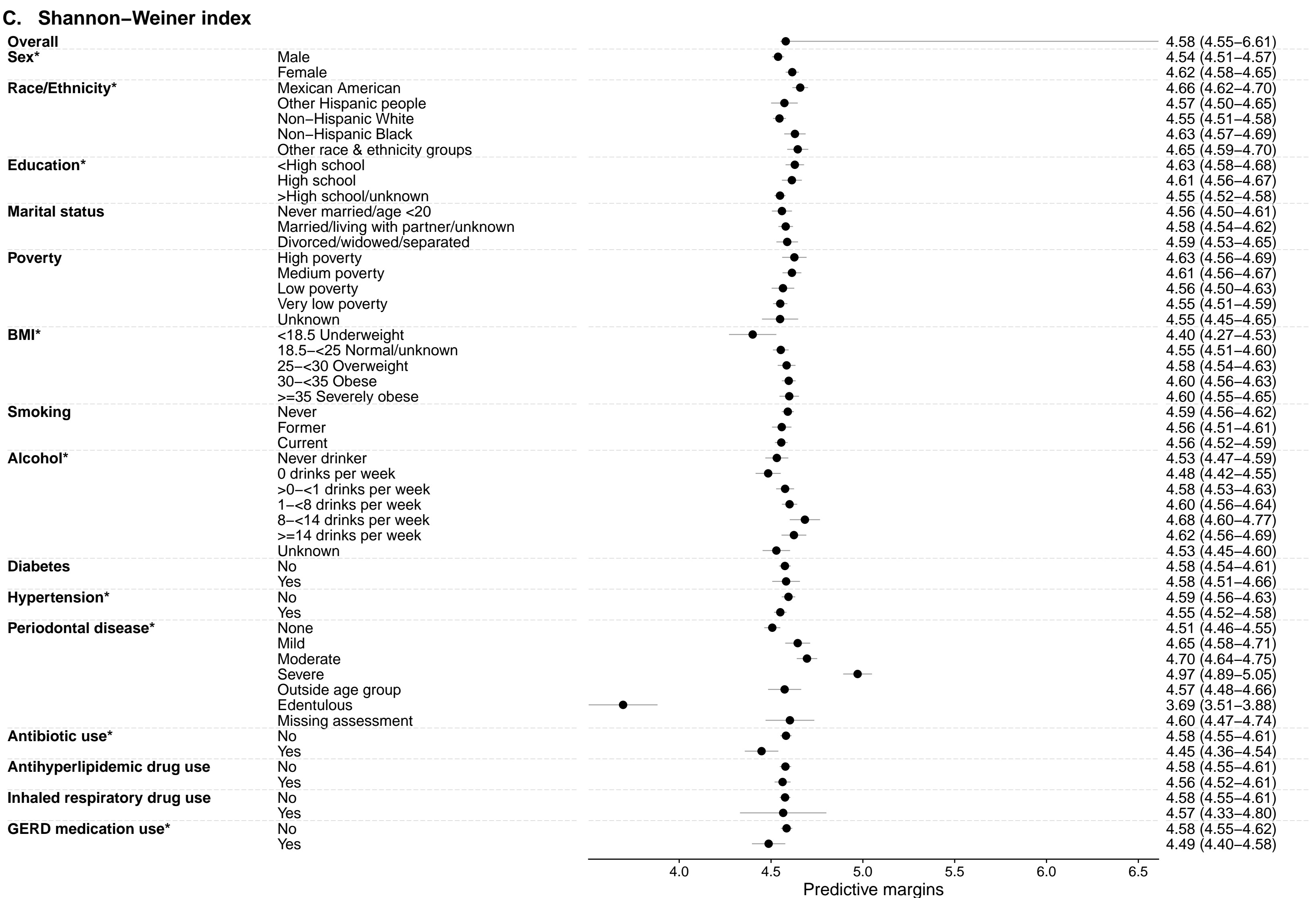

Supplement: Supplement 16 [file media-16.pdf]

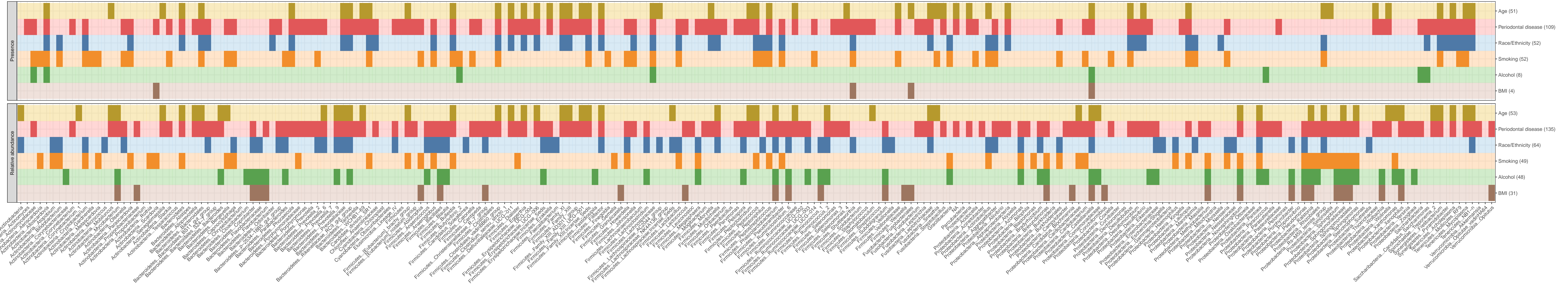

Supplement: Supplement 17 [file media-17.pdf]
